# Overexpression of IRS-4 Correlates with Procaspase 3 Levels in Tumoural Tissue of Patients with Colorectal Cancer

**DOI:** 10.1155/2018/3812581

**Published:** 2018-10-16

**Authors:** Patricia Sanmartín-Salinas, Luis G. Guijarro

**Affiliations:** ^1^Department of System Biology, Unit of Biochemistry and Molecular Biology, University of Alcalá, Alcalá de Henares, Spain; ^2^Centro de Investigación Biomédica en Red de Enfermedades Hepáticas y Digestivas (CIBEREHD), Spain

## Abstract

We reported that insulin receptor substrate 4 (IRS-4) levels increased in tissue from colorectal cancer (CRC) patients and promoted retinoblastoma-cyclin-dependent kinase activation. The aim of the present study was to evaluate the effect of IRS-4 on IGF-1 receptor pathway and its impact on procaspase 3 and PARP expression in RKO and HepG2 cancer cell lines. The results obtained* in vitro* were compared with those obtained from biopsies of patients with CRC (n = 18), tubulovillous adenomas (TA) (n = 2) and in matched adjacent normal colorectal (MANC) tissue (n = 20). IRS-4 overexpression in cultured cells induced the overactivation of IGF-1/BRK/AKT/GSK-3/*β*-catenin/cyclin D1 pathways, which led to increased expression of procaspase 3 and PARP protein levels. Studies carried out on CRC and TA tissues revealed the overactivation of the IGF-1 receptor signalling pathway, as well as the overexpression of procaspase 3 and PARP in tumoural tissue with respect to MANC tissue. The upregulation of IRS-4 in tumoural samples correlated significantly with the increase in pIGF-1 receptor (Tyr 1165/1166) (r = 0.84; p < 0.0001), procaspase 3 (r = 0. 77; p < 0. 0005) and PARP (r = 0. 89; p < 0. 0005). Similarly, we observed an increase in the proteolysis of procaspase 3 in tumoural tissue with respect to MANC tissue, which correlated significantly with the degradation of PARP (r = 0.86; p < 0.0001), p53 (r = 0.84; p < 0.0001), and GSK-3 (r = 0.78; p < 0.0001). The stratification of patient samples using the TNM system revealed that procaspase 3 and caspase 3 increased gradually with T values, which suggests their involvement in the size and local invasion of primary tumours. Taken together, our findings suggest that IRS-4 overexpression promotes the activation of the IGF-1 receptor pathway, which leads to the increase in procaspase 3 levels in CRC.

## 1. Introduction

Colorectal cancer (CRC) is one of the most prevalent cancers and is a frequent cause of cancer-related death [1]. A major determinant of CRC promotion, progression, and drug-resistance is the stimulation of the insulin-like growth factor (IGF) system [2] and nuclear translocation of IGF-1 receptor [3]. However, the therapeutic value of IGF-1 receptor targeting is under debate [4]. Antibodies targeting the IGF-1 receptor, the VEGF, or the EGF receptor show promise* in vitro *assays, but when given as an adjunct to conventional chemotherapy only marginal improvement in CRC patient survival has been observed [4–6]. It is therefore important to identify the molecular causes of the lack of response to anti-IGF-1 receptor antibodies [2, 3]. The IGF-1 receptor is a tyrosine kinase that phosphorylates IRS-1 or IRS-2 scaffold proteins in their C-termini [7]. Phosphorylation of the adaptor proteins creates binding sites to other proteins and/or enzymes, leading to activation of protein kinase B (AKT) and extracellular signal-regulated kinase (ERK) [8]. IRS-1 and IRS-2 are required for normal growth and glucose metabolism and accordingly are ubiquitously expressed [9].

Unlike the other members of the family, IRS-4 seems to have its own specific signalling pathway. In fact, IRS-4 was able to activate ERK in a PKC-dependent manner [10] or activate the PI3K pathway constitutively, even in the absence of extracellular ligands [11, 12]. This characteristic has been explained in part by the absence of binding sites in IRS-4 for the tyrosine phosphatase SHP-2, an enzyme that mediates the inhibitory feedback loop necessary in normal cell cycle regulation by IGF-1 [12]. Tumour growth and poor prognosis of cancer are strongly associated with sustained activation of the IGF-1 receptor signalling pathway [13, 14].

IRS-4 is increasingly associated with cancer pathogenesis and hyperplasia. Increased IRS-4 levels were found in the remnant liver after partial hepatectomy [15], as well as in uterine leiomyomas [16], subungual exostosis [17], breast cancer [12], hepatocellular carcinoma [18], leukaemia [19, 20], lung cancer [21], and colorectal cancer [22]. In recent times, the spectrum of cancer types in which IRS-4 is involved is increasing. In this context, the DNA sequencing studies of 7,416 human cancers of different tissular origin found very frequent deletions in cis-regulatory regions of the IRS-4 gene and it has been established as an oncogenic driver [12, 21]. Uncontrolled tumour cell proliferation is often characterized by aberrant activity of cell cycle proteins. We recently demonstrated that IRS-4 is involved in retinoblastoma-cyclin-dependent kinase (Rb-CDK) pathway activation in human CRC [23]. However, the signalling mechanism of IRS-4 in CRC remains unclear. Thus, the aim of the present study was to investigate the role of IRS-4 in the IGF-1 receptor signalling pathway in colon biopsies obtained from CRC patients and in cultured cells. Taking into account the role of the above-mentioned signalling pathways in apoptosis, we have analysed both procaspase 3 and PARP in the same samples.

## 2. Materials and Methods

### 2.1. Patients and Sample Collection

Tissue samples were collected from surgical resection specimens in 18 CRC and 2 tubulovillous adenoma (TA) patients at Hospital Universitario Principe de Asturias between 2010 and 2011. Immediately after the surgery, the tumours were staged by the pathologists of the Hospital. The TNM staging system proposed by the American Joint Committee on Cancer (AJCC) was used. The clinicopathological data, gender, and age of patients are summarized in Table S1 in Supplementary Materials. Tumour samples and matched adjacent normal colorectal (MANC) tissues were fresh frozen in liquid nitrogen immediately after removal and stored at - 80°C until use. This study was approved by the Institutional Ethic Committee of the Principe de Asturias Hospital and it has been conducted in accordance with the ethical standards established in the 1964 Declaration of Helsinki and its later amendments. Informed consent for the use of biopsies for research purposes was obtained from all patients prior to their inclusion in the study.

### 2.2. Cell Culture and Incubation Conditions

HepG2 and RKO cells obtained from ATCC were cultured in minimal essential medium (MEM) supplemented with 10% fetal bovine serum and 1% antibiotic/antimycotic solution. They were maintained in a humidified 37°C incubator in presence of 5% CO_2_. In the immunoprecipitation experiments, RKO cells were starved for 72 h and stimulated with IGF-1 (25 nM) for 30 minutes. RKO cells were treated with wortmannin (200 nM) during 18 h in some experiments. Cells were washed in ice-cold phosphate-buffered saline (PBS) and disrupted with lysis buffer for further analysis.

### 2.3. Transfection Assays

Cells overexpressing IRS-4 were obtained as previously described [23] with minor modifications. Briefly, DNA was isolated from RKO cells using DNeasy Blood & Tissue kit (Qiagen). Full-length IRS-4 DNA (3881 bp) was amplified by nested PCR using Pwo DNA polymerase (Roche). The resulting DNA fragments were ligated into the HindIII and EcoR1 sites of pcDNA3.1. The fidelity of the recombinant plasmid was assessed by DNA sequencing. Transfection of RKO and HepG2 cell with pcDNA3.1-IRS-4, herein after pcDNA (IRS-4) was performed using TurboFect (Thermo Scientific) according to the manufacturer's instructions. The empty pcDNA3.1 vector was used as a negative control. Stable transfectants were obtained after selection with G418 for different periods of time.

### 2.4. Quantitative PCR (qPCR)

qPCR was performed as previously described [21]. Total RNA was isolated using RNeasy Mini Kit (Qiagen) according to manufacturer's protocol. Contaminating genomic DNA was eliminated using RNase-free DNase (Qiagen). Total RNA (2*μ*g) was reverse transcribed into single-stranded cDNA using the AMV First Strand cDNA synthesis kit (Roche) according to the manufacturer's instructions. Real-time PCR amplification reactions were performed using the SYBR Green PCR Master Mix (Applied Biosystems). The cycling conditions and the primers used to amplify IRS-1, IRS-4, and 18S have been described previously [18].

### 2.5. Protein Extraction

RKO and HepG2 cells were washed in ice-cold phosphate-buffered saline (PBS) and disrupted with ice-cold lysis buffer containing (Tris-HCl 50 mM, pH 7.4, EDTA 5 mM, EGTA 1 mM, PMSF 1 mM, leupeptin 5 *μ*g/ml, and aprotinin 5 *μ*g/ml). Then cells were disrupted by sonication and centrifuged (100.000 g for 30 min at 4°C) for further analysis. Frozen human colorectal tissues were homogenized in ice-cold lysis buffer by mechanical disruption. To remove connective tissue a centrifugation (660 rpm for 10 minutes at 4°C) was carried out. The supernatant was collected and centrifuged again (20.000 rpm for 30 min at 4°C). The pellet was then resuspended with ice-cold lysis buffer plus Triton X-100 (1%). The purified fraction was obtained by centrifugation (20.000 rpm for 15 min 4°C) and stored at -80°C until use. The amount of protein was determined using a Bradford protein assay kit (Bio-Rad).

### 2.6. Immunoprecipitation and Immunoblotting

For immunoprecipitation (IP), 500 *μ*g of total protein resulting from cell or tissue extracts was incubated overnight at 4°C with 2 *μ*g of specific antibodies against IRS4 (Santa Cruz Biotechnology), IRS-1 (Santa Cruz Biotechnology), or BRK (Santa Cruz Biotechnology). Thereafter, protein G-agarose beads were added, followed by incubation for 2 h at 4°C. Negative controls (C-) were performed replacing the colonic sample or lysis extract with buffer and maintaining all other reagents used in the immunoprecipitation protocol. After washing three times with ice-cold lysis buffer, the immunocomplexes were analysed by Western Blotting (WB) as previously described [24]. Briefly, the whole lysate or immunoprecipitated materials were resolved in SDS-PAGE and transferred to nitrocellulose membranes overnight at 25 V and 4°C. Bands were detected with ECL™ Western Blotting Analysis System. Quality control of the protein extracts were performed by SDS-PAGE and subsequent staining using Coomassie Blue, as previously described [25]. Accurate and normalized densitometric data was achieved by producing a twofold dilution series of the protein lysate to ensure that the amount of lysate loaded was within the linear dynamic range [25]. Intensity of blot bands obtained from human samples was estimated by densitometry using Scion Image software (Scion Corporation, version beta 4.0.2, USA). The relative amount of each protein was normalized using p66 protein which did not change significantly during pathological conditions.

### 2.7. Statistical Analysis

Total or phosphorylated proteins expression levels among CRC and MANC tissues were compared using paired Student's t-test. When the patients were stratified following the TNM staging system, the statistical differences of the biochemical parameter between subgroups were analysed by ANOVA test followed by Bonferroni correction. The correlations between IRS-4 or IRS-1 expression levels and the IGF-1 receptor pathway proteins in patients with CRC were analysed by Pearson's correlation coefficient (r). For every patient (n=20), we obtained the difference (∆) between the densitometric value of each protein (total or phosphorylated) in the CRC and MANC sample. At least three independent experiments were performed to obtain each result. The levels of significance were set at p < 0.05 (*∗*), p < 0.01 (*∗∗*), p < 0.001 (*∗∗∗*), and p < 0.0001 (*∗∗∗∗*).

## 3. Results

### 3.1. Effect of IRS-4 Overexpression on Signalling Pathways and in Biomarkers Involved in Proliferation and Apoptosis in RKO and HepG2 Cells

**Figure 1:**
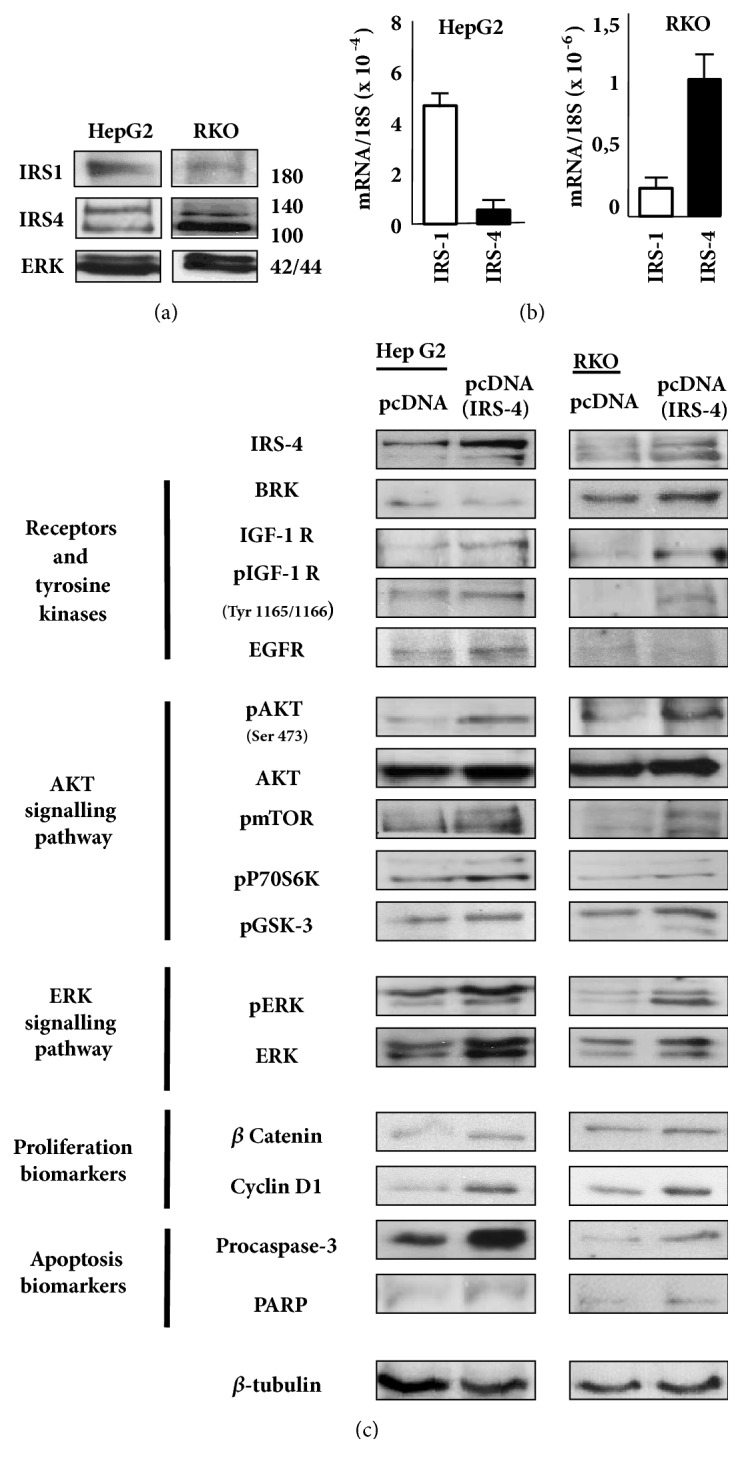
(a) Western blot analyses of IRS-4, IRS-1, and ERK levels in RKO and HepG2 cell lines. (b) IRS-1 and IRS-4 mRNA expression levels in RKO and HepG2 cell lines were detected using qPCR; graphs depict the calculated ratios of IRS-1/IRS-4 to 18S for each sample. (c) RKO and HepG2 cells were transiently transfected with pcDNA (IRS-4) or pcDNA (negative control); cells were lysed and several proteins were analysed by western blot. A representative experiment of three performed is shown.

We evaluated IRS-1 and IRS-4 expression levels by western blot (protein levels) and pPCR (mRNA levels) in HepG2 and RKO cells. RKO cells showed high IRS-4 and low IRS-1 expression levels when analysed using both techniques. In contrast, HepG2 cells showed high levels of IRS-1 and low levels of IRS-4 (Figures 1(a) and 1(b)).

We overexpressed IRS-4 in HepG2 and RKO cells to check the impact of the protein on the IGF-1 signalling pathway and its relationship to proliferation/apoptosis biomarkers in both cell types (Figure 1(c)).

The upregulation of IRS-4 levels induced the activation of the IGF-I receptor pathway and increased the phosphorylation of AKT, mTOR, p70S6 kinase, GSK-3 and ERK in both cell types (Figure 1(c)). The activation of the complete pathway increased the levels of *β*-catenin and cyclin D1 (two biomarkers of cell proliferation) in both cell types studied. The overexpression of IRS-4 increased the levels of procaspase 3 and PARP in both cell types, although this effect was more pronounced in HepG2 than in RKO cells, which may be due to the differences in the basal levels of expression of IRS-1 and IRS-4 in both cell types.

### 3.2. Study of the Mechanism Involved in the Effect of IRS-4 on IGF-1 Receptor Signalling Pathways in RKO Cells

The following set of experiments was performed on RKO cells solely with the aim of translating the data obtained in vitro to the study in vivo using samples from CRC patients.

**Figure 2:**
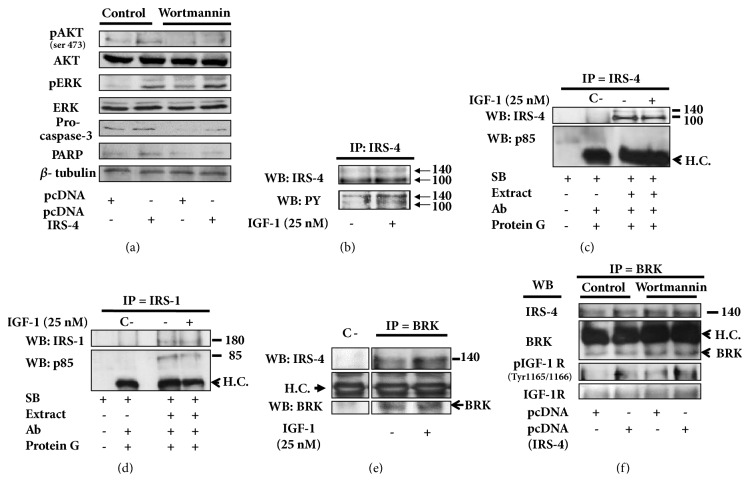
(a) RKO cells stable-transfected with pcDNA (IRS-4) or pcDNA were untreated or treated with wortmannin (200 nM) during 18 h and several proteins were analysed by western blot. (b) RKO cells were starved overnight with serum-free medium and stimulated with IGF-1 (25 nM) during 30 min, then harvested, and lysed. Immunoprecipitation with anti-IRS-4 antibody was carried out and tyrosine phosphorylation was analysed by immunoblot. (c) Immunoprecipitations with anti-IRS-4 antibody and (d) anti-IRS-1 antibody was performed in RKO cells after IGF-1 treatment (25 nM) during 30 min, then the association with *α*p85 was studied by immunoblot. (e) RKO cells with or without IGF-1 stimulation (25 nM) during 30 min were lysed and immunoprecipitated with anti-BRK antibody and its interaction with IRS-4 was studied by western blot. (f) RKO cells stable-transfected with pcDNA (IRS-4) or pcDNA and treated with wortmannin (200 nM) during 18 h were immunoprecipitated with anti-BRK antibody and its association with IRS-4, IGF-1 receptor and phosphorylated IGF-1 receptor were studied by western blot. Negative control (C-) was assessed replacing the lysis extract for sample buffer. Results shown are representative for two or three independent experiments. H.C. = heavy chain of the immunoprecipitation antibody. SB = sample buffer.

The activation of AKT pathways by IRS-4 in RKO cells was dependent on PI3K, since the treatment of the cells with wortmannin (200 nM) for 18 h inhibited specifically AKT phosphorylation without affecting ERK phosphorylation. The specific inhibition of PI3K-AKT pathways decreased the levels of procaspase 3 and PARP (Figure 2(a)). With the aim of studying the mechanism involved in the effect of IRS-4 on IGF-1 signalling pathways, we stimulated RKO cells with IGF-1 (25 nM) for 30 min. The activation of IRS-4 by IGF-1 was assessed by immunoprecipitation of IRS-4 followed by immunoblotting with anti-PY99 antibody. We observed an increase in tyrosine phosphorylation of IRS-4 after IGF-1 stimulation (Figure 2(b)). However, the increase in the phosphorylation of IRS-4 did not correlate with its association to *α*p85. In fact, after immunoprecipitation of IRS-4 and subsequent immunoblot against *α*p85, the physical interaction between the two proteins was not observed, either under basal conditions or following stimulation with IGF-1 (Figure 2(c)). When we carried out the immunoprecipitation of IRS-1 we observed, as expected, the association of the scaffold protein with *α*p85, both in basal and IGF-1 stimulated cells (Figure 2(d)). Interestingly, when BRK was immunoprecipitated, we detected the formation of the complex between BRK and IRS-4 (Figure 2(e)). The amount of IRS-4 bound to BRK increased after IGF-1 stimulation (Figure 2(e)). In addition, we observed the presence of phosphorylated IGF-1 receptor (Tyr 1165/1166) in BRK immunoprecipitated proteins (Figure 2(f)). The amount of phosphorylated IGF-1 receptor increased when IRS-4 was overexpressed and did not change after treatment with wortmannin (200 nM) for 18 h, which suggests that the formation of this ternary complex is regulated upstream of PI3 kinase (Figure 2(f)).

### 3.3. Upregulation of IRS-4 and IGF-1 Receptor Signalling Pathways in Colorectal Cancer Tissue and Its Relationship with Biomarkers Involved in Proliferation and Apoptosis

**Figure 3:**
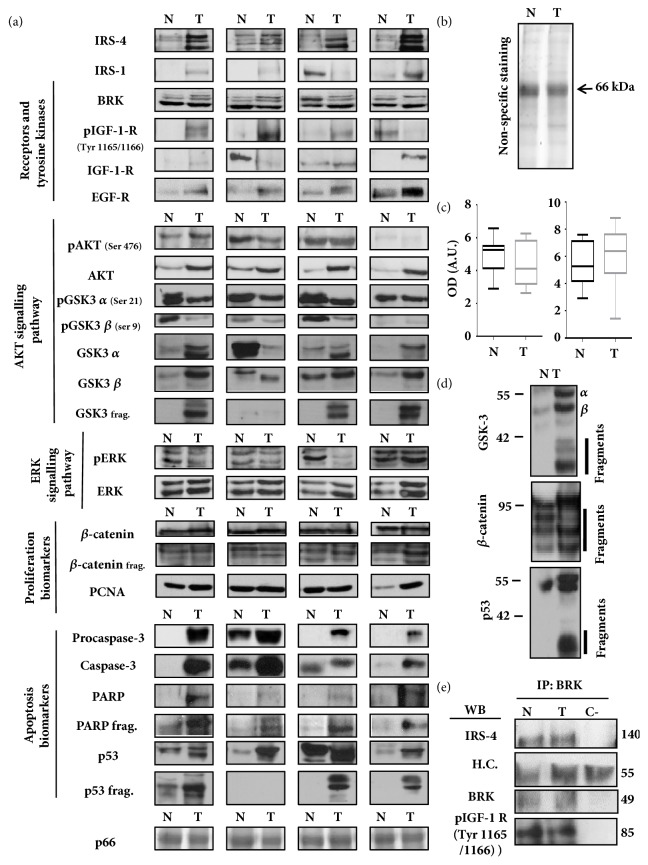
(a) Immunoblot of IRS-4, IGF-1 signalling axis proteins and proliferation and apoptosis biomarkers in human colorectal carcinoma tissues (T) and matched adjacent normal colorectal tissues (N). The western blot was performed in normal (n=20) and tumoural (n=20) colorectal samples, results obtained from four representative patients are shown out of 20 subjects studied. (b) Nonspecific staining performed with Coomassie Blue (CB) of all samples was used as loading and quality control. CB staining of a representative patient is shown in normal (N) and tumoural (T) tissue. (c) The graph bar shows the mean ± SD of the densitometric analysis of the 66 kDa band and PCNA from normal (n=20) and tumoural (n=20) colorectal samples analysed by CB staining and western blot respectively. (d) Representative western blot of GSK-3, *β*-catenin and p53 fragments observed in normal (N) and tumoural (T) tissues. Results obtained from one representative patient are shown out of 20 subjects studied. (e) Immunoprecipitation using anti-BRK antibody and subsequent analysis of BRK, IRS-4 and phospho-IGF-1 receptor in samples from normal (N) and tumoural (T) tissues. The immunoprecipitation was performed three times for each of 6 selected patients' samples. Results obtained from one representative patient are shown. Sample buffer was replaced for lysis extract in negative control (C-). H.C. = heavy chain of the immunoprecipitation antibody. N = MANC tissue. OD = optical density. A.U. = arbitrary units.

We studied the expression levels by immunoblot of IRS-4 and IGF-1 receptor signalling pathway proteins and proliferation/apoptosis biomarkers in 20 surgically resected tumours and in 20 MANC tissue samples from the patients described in Table S1 in Supplementary Materials. As a point of reference, we studied the levels of EGF receptor and PCNA, two proteins involved in colon carcinogenesis. The densitometric analysis showed interindividual variations both in tumoural and in MANC tissue samples of CRC patients. We show the results of four patients that represent this heterogeneity (Figure 3(a)). We also studied, in the same samples, the biomarkers involved in proliferation (*β*-catenin and PCNA) and in apoptosis (procaspase 3, caspase 3, PARP, PARP fragment, p53, and p53 fragment) (Figure 3(a)).

Figure 3(b) shows a Coomassie Blue staining of a representative patient. The band of 66 kDa (p66) corresponds to a very abundant protein in colonic extract, as yet to be identified. The densitometric analysis of p66 in 20 CRC and 20 MANC samples shows insignificant differences between normal and tumoural samples (Figure 3(c)). We did not observe statistical differences in PCNA levels between normal and tumoural samples (Figure 3(c)). In the population study, we observed proteolytic fragments of GSK-3, *β*-catenin, and p53 (Figure 3(d)), which have been estimated by densitometry.

The study of the physical association between BRK, IRS-4, and IGF-1 receptor as previously observed in RKO cells was carried out through specific immunoprecipitation of BRK in human CRC and MANC samples (Figure 3(e)). IRS-4 and phosphorylated IGF-1 receptor coprecipitated with BRK in CRC and MANC tissues. However, we did not observe differences in the amount of the components of the ternary complex between tumoural and normal samples (Figure 3(e)).

**Figure 4:**
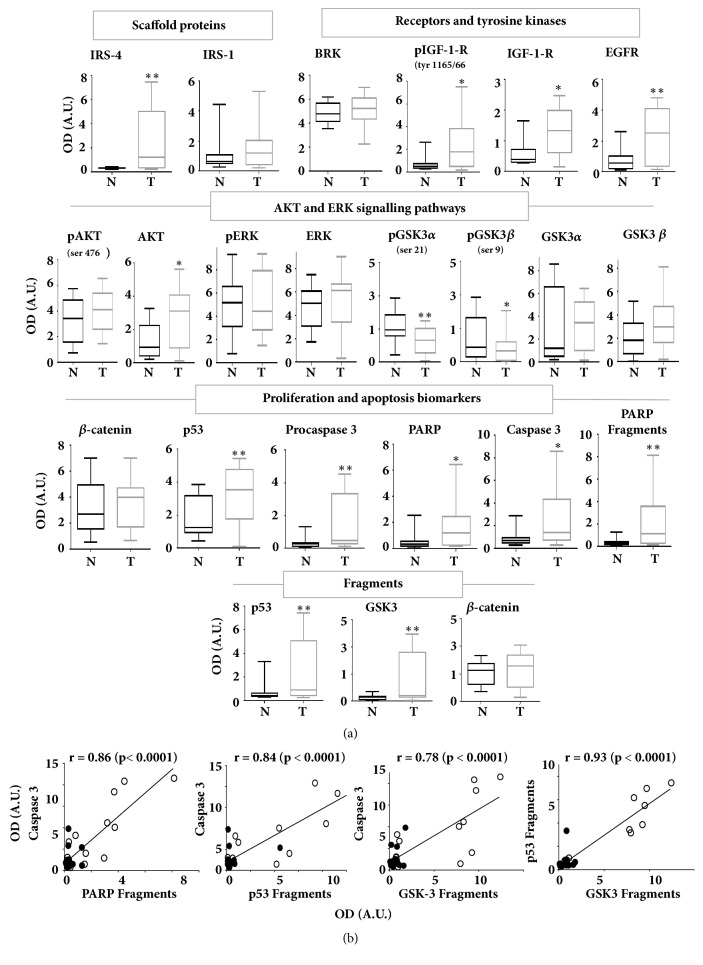
(a) Bar graphs obtained by densitometric analysis of western blot data of IRS-4, IGF-1 signalling axis proteins, and proliferation and apoptosis biomarkers in human colorectal carcinoma tissues (T) (n=20) and matched adjacent normal colorectal tissues (N) (n=20). Results represent the mean ± SD. Levels of significance: *∗* p<0.05; *∗∗*p<0.01. (b) Pearson's correlation coefficient between caspase 3 and PARP fragments, p53 fragments and GSK-3 fragments and between p53 fragments and GSK-3 fragments of CRC and MANC tissues of the 20 patients studied. The black and white points correspond to MANC and CRC tissues, respectively. N = MANC tissue. OD = optical density. A.U. = arbitrary units.

The data from the densitometric quantification of the immunoblotted proteins shown in Figure 3(a) were statistically analysed for the 20 CRC patients (Figure 4(a)). The results revealed a significant increase in IRS-4, but not in IRS-1, in CRC tissue with respect to MANC tissue (Figure 4(a)). The changes in IRS-4 were accompanied by the increase in phosphorylated IGF-1 receptor (Tyr 1165/1166), in IGF-1 receptor levels, and in EGF receptor levels, but not in BRK levels. The increase in IRS-4 affected AKT levels slightly, but did not change the ERK signalling pathways. The levels of GSK-3/*β*-catenin system did not change during carcinogenic process. In contrast, we observed a reduction of GSK-3*α* and GSK-3*β* phosphorylation in tumour tissue with respect to normal tissue, which indicates an activation of these enzymes. The increase in tyrosine kinase receptors for IGF-1 and EGF, together with the absence of the increase in the phosphorylation of downstream kinases (pAKT, pERK, and pGSK-3), suggests the uncoupling of both signalling pathways in CRC.

As we have observed in HepG2 and RKO, to a lesser extent, the overexpression of IRS-4 in tumoural tissue was accompanied by the increase in procaspase 3 and PARP with respect to normal tissue. We observed also a slight increase in p53 in CRC tissue with respect to MANC tissue. The increase in the above-mentioned proteins in tumoural tissue with respect to MANC tissue was accompanied by their fragmentation, which suggests a general proteolytic activation during CRC. However, we did not observe any changes between both normal and tumoural tissue in native *β*-catenin levels or in fragmented *β*-catenin (Figure 4(a)).

To establish the possible relationships between the biochemical parameters analysed, we followed the Pearson's correlation coefficient method. As expected, the amount of caspase 3 fragments correlated significantly with PARP fragments (r = 0.86; p < 0.0001) in CRC and MANC tissues of the 20 patients studied (Figure 4(b)). Interestingly, we also observed a strong correlation between caspase 3 levels and p53 fragments (r = 0.84; p < 0.0001) and between caspase 3 and GSK-3 fragments (r = 0.78; p < 0.0001) in the same samples (Figure 4(b)). All of these results are logical, given that PARP, p53 and GSK-3 are caspase 3 substrates. Additionally, we identified a very strong correlation between GSK-3 fragments and p53 fragments (r = 0.93; p < 0.0001) in the same samples. The white and black points correspond to CRC and MANC tissues, respectively (Figure 4(b)).

To study in more detail the possible association between the increase in IRS-4 and the above-mentioned biochemical parameters in CRC, we calculated the Pearson's coefficient between Δ IRS-4 and the corresponding proteins involved in the IGF-1 receptor signalling cascade and in apoptosis biomarkers from the 20 patients described in Table S1 in Supplementary Materials. We used the same method for IRS-1. The results are shown in Table 1.

The increase in IRS-4 expression levels observed in CRC samples correlated positively with those corresponding to phosphorylated IGF-1 receptor (tyr 1165/1166) (r=0.84; p<0.0001), AKT (r= 0.69; p<0.002), procaspase 3 (r= 0.77; p<0.0005) and PARP (r= 0,89; p<0.000005).

Using the same method, the correlation of ΔIRS-1 and the same set of proteins showed marginally significant results in CRC samples (Table 1).

### 3.4. Levels of IRS-4, IGF-1 Receptor Signalling Pathway Proteins, and Apoptosis Biomarkers in Tumours from CRC Patients Stratified by TNM Staging

The densitometric quantification of each protein was used to assess the possible importance of the above-mentioned proteins on clinicopathological features of CRC as determined by TNM staging.

**Figure 5:**
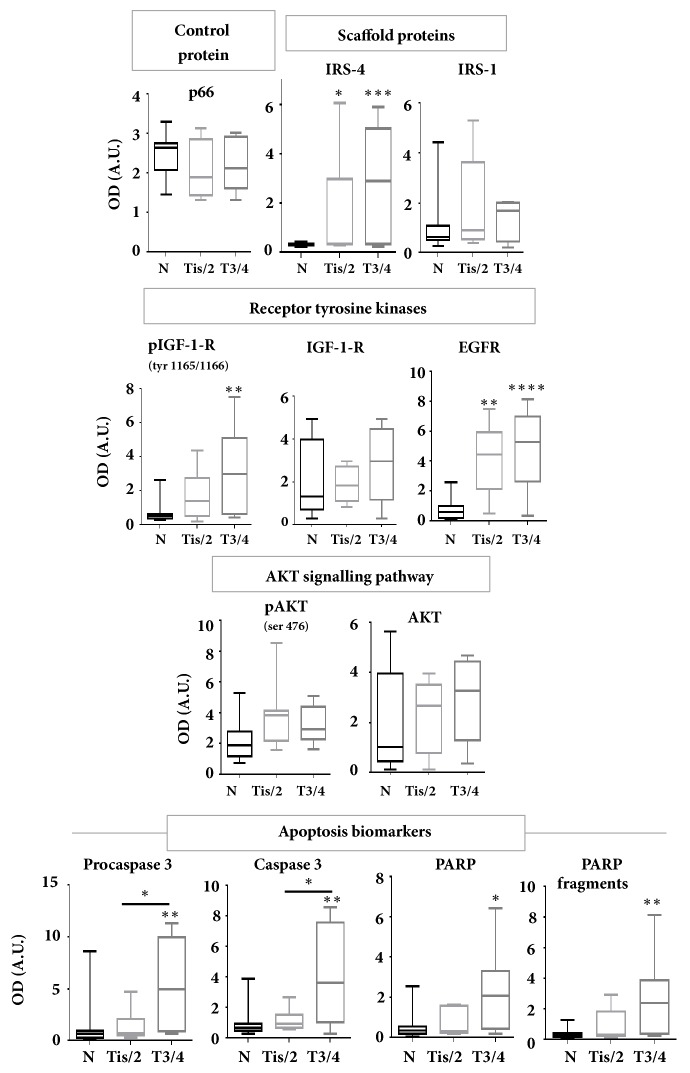
The 20 patients were stratified into different stages of T (extent of the primary tumour) of TNM classification and densitometric quantification of western blots from all patients was represented in bar graphs. Results represent the mean ± SD. Levels of significance: *∗* p<0.05; *∗∗*p<0.01; *∗∗∗* p<0.001. N = MANC tissue. OD = optical density. A.U. = arbitrary units.

In this part of the study, our attention was focused only on the proteins that correlate with ∆IRS-4 with a Pearson's correlation coefficient of r > 0.7. We included also canonical components of the IGF-1 and EGF signalling pathways, as IRS-1, IGF-1 receptor, EGF receptor, pAKT and AKT for comparative purposes. CRC samples were stratified in two groups: Tis/T2 and T3/T4. Both groups were compared with MANC samples. The results of the statistical analysis of the above-mentioned immunoblotted proteins are shown in Figure 5. We observed a gradual increase in the levels of IRS-4, phosphorylated IGF-1 receptor, EGF receptor, procaspase 3 and PARP following the increment in the T value (Figure 5). We must keep in mind that T value represents the size and nearby invasion of primary tumour. Other important molecules involved in the IGF-1 signalling pathway, such as IRS-1, IGF-1 receptor, pAKT and AKT did not change significantly in relation to the size of the tumour (Figure 5). We also observed the gradual increase in caspase 3 and the fragments corresponding to PARP, p53 and GSK-3 in relation to T value. Interestingly, we identified a significant increase in procaspase 3, as well as in caspase 3, in T3/T4 group with respect to Tis/T2 group (Figure 5).

**Figure 6:**
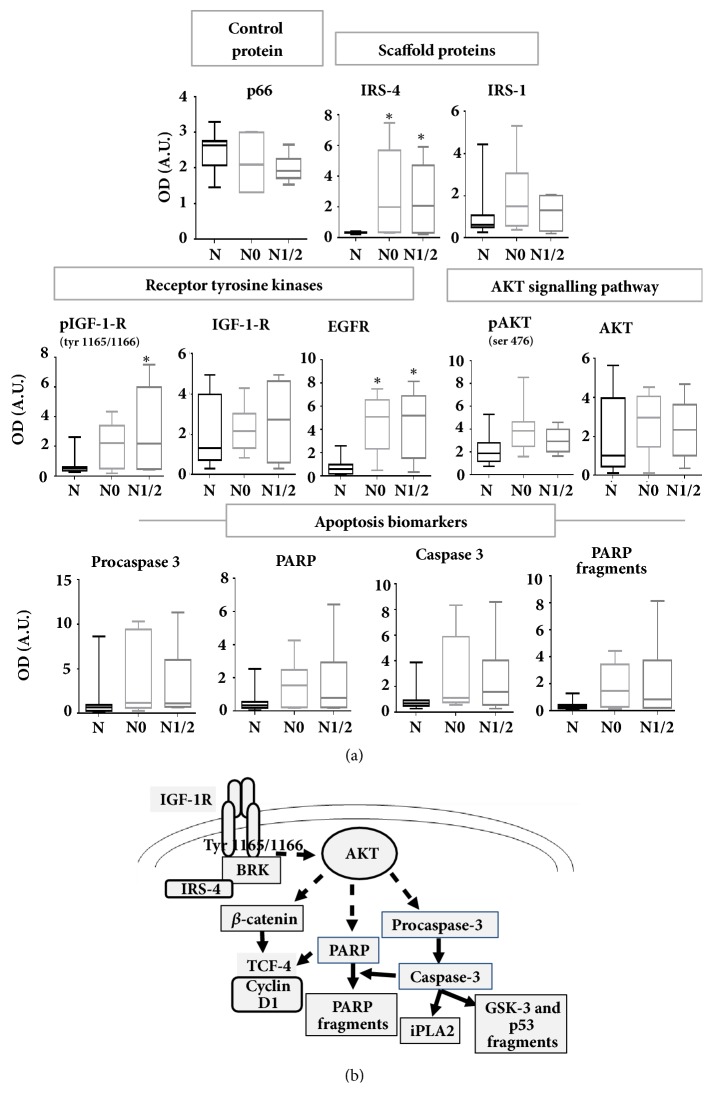
(a) The 20 patients were stratified into stage N0 (absence of regional lymph node metastasis) or N1/2 (presence of regional lymph node metastasis) of TNM classification and densitometric quantification of western blots from all patients was represented in bar graphs. Results represent the mean ± SD. Levels of significance: *∗* p<0.05; *∗∗*p<0.01; *∗∗∗* p<0.001. (b) Schematic model on the role of IRS-4 on CRC. N = MANC tissue. OD = optical density. A.U. = arbitrary units.

CRC samples were also stratified in two groups corresponding to lymph node cancer staging—N0 (free of lymphatic metastasis) or N1/N2 (at least one lymphatic metastasis)—and were compared with MANC samples. The same proteins were studied by immunoblot and densitometric analysis in the stratified groups. We observed a gradual increase in the phosphorylated IGF-1 receptor according to the increment in N value. However, IRS-4 and EGF receptor increased in the same proportion in N0 and in N1/2 groups (Figure 6). IRS-1, IGF-1 receptor, pAKT and AKT levels did not change significantly between N0, N1/2 and MANC groups (Figure 6). We used p66 protein as lysate quality and loading control of electrophoresis (Figures 5 and 6).

## 4. Discussion

The tyrosine phosphorylation of IGF-1 receptor has been associated with the resistance of CRC patients to conventional therapies and targeted therapeutic agents [26, 27]. This phenomenon may be caused by the increase in IRS-4 levels in CRC tissue [23]. In the present study, we observed that IRS-4 overexpression in HepG2 and RKO cancer cells is associated with the activation of IGF-1 receptor signalling pathways in the absence of extracellular ligand. The effect of IRS-4 may be dependent on BRK, given that we observed the presence of ternary complexes between IRS-4/BRK and phosphorylated IGF-1 receptors in RKO cells and in samples from CRC tissue. The formation of this type of complexes was independent of PI3K because was observed in absence and in presence of wortmannin.

The potential importance of the formation of this class of ternary complexes resides in the fact that IRS-4 stimulates the tyrosine kinase activity of BRK [28], which in turn is able to activate IGF-1 receptor [29]. Moreover, we observed the increase in phosphorylation of Tyr 1165/1166 residues of IGF-1 receptor in RKO after IRS-4 overexpression, as well as after stimulation with IGF-1. Tyr 1165/1166 are located in the catalytic domain of the receptor tyrosine kinase and their phosphorylation is necessary for the complete activation of the IGF-1 receptor [30]. This mechanism could explain the constitutive PI3K/AKT pathway hyperactivation observed in the absence of grow factors when IRS-4 is overexpressed [11, 12]. The stimulation of this pathway was associated with the increase in anchorage-independent survival of mammary epithelial cells in culture [29] and possibly in metastatic dissemination [29]. Previously, we demonstrated that IRS-4 increased HepG2 [10] and RKO [23] proliferation by a Rb-CDK pathway. In addition we showed that overexpression of IRS-4 correlated with clinical staging in colorectal cancer patients [22].

In the present paper we show that the hyperactivation of IGF-1R/IRS-4 axis correlates with procaspase-3 overexpression in HepG2 and in RKO cells. This finding has been confirmed in human CRC samples. In very recent studies, procaspase 3 has been associated with CRC disease progression and overall survival [31], in part because its fragmentation during inflammation or after cancer therapy increases the levels of caspase 3 active form, which leads to constitutive activation of iPLA2 [32]. Phospholipase A2 activation results in the release of arachidonic acid, which is the substrate of COX-2, and as a consequence of this activity PGE2 is produced [33]. This last eicosanoid stimulates proliferation of cells through the Wnt/*β*-catenin signalling pathways [33] and inhibits apoptosis by stabilization of survivin-procaspase 3 complexes [34]. The effect of IRS-4 on procaspase 3 levels in RKO cells was dependent on PI3K because it was inhibited by wortmannin incubation. At present we do not fully understand the mechanism involved in this finding. However, it has been demonstrated that IRS-4 is a novel modulator of BMP4/Smad and AKT signalling during early muscle differentiation [35], and that BMP4 promotes cell survival through an increase in procaspase 3 and bcl-2 expression by a PI3K dependent mechanism in smooth muscle cells [36].

In addition, we have observed the close correlation between IRS-4 and PARP in HepG2 and RKO cells and in CRC samples. PARP is a coactivator of the *β*-catenin /TCF-4 complex [37]. In this respect, we observed the increase in *β*-catenin and the surrogate marker cyclin D1 in cells overexpressing IRS-4, which has been positively associated with the increase in G1 checkpoint cell cycle proteins in CRC samples [23]. Based on these observations, we propose that the regulatory role of IRS-4 on procaspase 3, PARP and cyclin D1 levels may be of some importance in the progression of CRC.

To further understand this last hypothesis, we studied the association of IRS-4 with the above-mentioned proteins in CRC patients stratified using the TNM system.

We observed the gradual increase in IRS-4, pIGF-1 receptor, EGF receptor, procaspase 3 and PARP, according to the severity of the disease, as measured by the TNM system. Moreover, the procaspase 3 and caspase 3 levels were higher in T3/T4 group than in Tis/2 group. In a previous study, Roy et al. have shown that procaspase 3 and caspase 3 are overexpressed in human colon carcinoma [38] and very recently Zhou et al. have observed that caspase-3 regulates the migration, invasion, and metastasis of colon cancer cells [39]. In clinical studies, low levels of caspase 3 predict favourable response to 5FU-based chemotherapy in advanced colorectal cancer [40]. Conversely, higher amounts of activated caspase 3 in the tumour predict worse treatment outcomes in patients with CRC [31]. The mechanism involved in these findings may be related to the increase in the autocrine factor PGE2 by a caspase 3 dependent mechanism, which is able to stimulate cell regrowth and angiogenesis in different types of human cancers [31, 32, 40]. In fact, caspase 3 overexpression is associated with unfavourable outcome to therapy in melanoma [41], and to cytotoxic therapy in head and neck squamous cell carcinoma [32] and in advanced breast cancer [32]. Our results, together with previous reports [42], suggest that in CRC tissue there is a general activation of the proteolytic enzymes. In fact, in tumoural tissue we observe a significant increase in the fragmentation of PARP, p53 and GSK-3. The first two are substrates of caspase 3 [43, 44]; however, the third, to our knowledge, is not.

A recent hypothesis that is gaining ground is that proteolysis is necessary in the process of tissue remodelling involved in cancer invasion. In this regard, the effect of IRS-4 on procaspase 3/caspase 3 expression levels may play a decisive role in tumour progression. Our hypothesis on the role of IRS-4 in CRC is depicted in Figure 6(b).

## 5. Conclusions

These results suggest that IRS-4 overexpression promotes IGF-1 receptor pathway activation through the formation of a ternary complex between IRS-4/BRK/pIGF-1R in RKO cells and in CRC samples leading to the increase in procaspase 3 levels. We also present evidence supporting that IRS-4 is upregulated in CRC tissue and its expression is positively associated with pIGF-1R, procaspase 3 and PARP which are also overexpressed in CRC and their levels increase according to the severity of the disease.

## Figures and Tables

**Table 1a:** (a) Correlations between ∆IRS-4/ ∆IRS-1 and ∆IGF-I pathway proteins in tumour and normal tissue

Δ IRS-4 vs	Δ IRS-1	Δ BRK	Δ pIGF-1R	Δ IGF-1R	Δ EGFR	Δ pAKT	Δ AKT	ΔGSK3*α*	ΔGSK3*β*	ΔpGSK3 *α* (ser 21)	ΔpGSK3 *β* (ser 9)	ΔGSK3 frag.
r	0,54	0,17	0,84	0,29	0,51	0,13	0,69	0,44	0,59	0,32	0,03	0,87
95% confidence interval	0,06 to 0,82	-0,55 to 0,74	0,58 to 0,94	-0,23 to 0,69	0,03 to 0,81	-0,38 to 0,59	0,30 to 0,88	-0,07 to 0,77	0,14 to 0,84	-0,20 to 0,70	-0,46 to 0,52	0,66 to 0,95
R square	0,29	0,027	0,70	0,08	0,26	0,01	0,48	0,19	0,35	0,10	0,001	0,76
P (one-tailed)	0,014	0,334	< 0,0001	0,132	0,019	0,304	0,001	0,086	0,014	0,222	0,892	< 0,0001
P value summary	*∗*	ns	*∗∗∗∗*	ns	*∗*	ns	*∗∗*	ns	*∗*	ns	ns	*∗∗∗∗*

Δ IRS-1 vs	Δ IRS-4	Δ BRK	Δ pIGF-1R	Δ IGF-1R	Δ EGFR	Δ pAKT	Δ AKT	ΔGSK3*α*	ΔGSK3*β*	ΔpGSK3 *α* (ser 21)	ΔpGSK3 *β* (ser 9)	ΔGSK-3 frag.

r	0,54	0,12	0,40	0,60	0,40	0,47	0,55	0,67	0,28	0,08	-0,06	0,31
95% confidence interval	0,06 to 0,81	-0,58 to 0,72	-0,10 to 0,75	0,16 to 0,84	-0,10 to 0,75	-0,02 to 0,78	0,07 to 0,82	0,27 to 0,87	-0,25 to 0,68	-0,42 to 0,55	-0,54 to 0,44	-0,21 to 0,70
R square	0,29	0,01	0,17	0,37	0,17	0,22	0,30	0,46	0,08	0,007	0,004	0,1
P (one-tailed)	0,014	0,375	0,058	0,006	0,057	0,031	0,013	0,004	0,293	0,757	0,802	0,230
P value summary	*∗*	ns	ns	*∗∗*	ns	*∗*	*∗*	*∗∗*	ns	ns	ns	ns

**Table 1b:** (b) Correlations between ∆IRS-4/∆IRS-1 and ∆ERK, ∆*β* Catenin and ∆ apoptosis biomarkers in tumour and normal tissue

Δ IRS-4 vs	Δ pERK	Δ ERK	*β*-Catenin	*β*-Catenin frag.	Δ PCNA	Δ Procaspase 3	Δ Caspase3	Δ PARP	Δ PARP frag.	Δp53	Δp53 frag.
r	0,11	0,56	0,57	0,19	0,23	0,77	0,64	0,89	0,78	0,33	0,87
95% confidence interval	-0,40 to 0,57	0,08 to 0,82	0,10 to 0,83	-0,33 to 0,63	-0,51 to 0,77	0,44 to 0,91	0,21 to 0,86	0,69 to 0,96	0,47 to 0,92	-0,19 to 0,70	0,67 to 0,95
R square	0,01	0,31	0,32	0,04	0,05	0,58	0,41	0,79	0,61	0,10	0,77
P (one-tailed)	0,339	0,012	0,021	0,464	0,271	0,0003	0,0036	< 0,0001	0,0002	0,212	<0,0001
P value summary	ns	*∗*	*∗*	ns	ns	*∗∗∗*	*∗∗*	*∗∗∗∗*	*∗∗∗*	ns	*∗∗∗∗*

Δ IRS-1 vs	Δ pERK	Δ ERK	*β*-Catenin	*β*-Catenin frag	Δ PCNA	Δ Procaspase 3	Δ Caspase 3	Δ PARP	Δ PARP frag.	Δ p53	Δ p53 frag.

r	0,14	0,02	0,21	-0,46	0,24	0,11	-0,07	0,31	0,06	0,27	0,20
95% confidence interval	-0,37 to 0,59	-0,48 to 0,51	-0,31 to 0,64	-0,78 to 0,04	-0,50 to 0,78	-0,40 to 0,57	-0,55 to 0,43	-0,21 to 0,70	-0,44 to 0,54	-0,25 to 0,67	-0,32 to 0,63
R square	0,02	0,0004	0,04	0,21	0,06	0,01	0,005	0,10	0,004	0,07	0,04
P (one-tailed)	0,292	0,470	0,421	0,070	0,262	0,341	0,390	0,116	0,405	0,297	0,454
P value summary	ns	ns	ns	ns	ns	ns	ns	ns	ns	ns	ns

The association between IRS-4/IRS-1 and the above-mentioned biochemical parameters in CRC was analysed by Pearson's correlation coefficient (r). ∆ = difference between the protein expression in matched adjacent normal colonic tissue (n=20) and in CRC tissue (n=20). Significance levels: *∗* p < 0.05; *∗∗* p < 0.01; *∗∗∗* p < 0.001; *∗∗∗∗* p < 0.0001.).

## Data Availability

The clinical data used to support the findings of this study were provided by the Hospital Universitario Principe de Asturias. Access to clinical data will be considered by the author upon request, with permission of the director of this hospital. The in vitro data used to support the findings of this study are available from the corresponding author upon request

## Supplementary Materials

Supplementary Materials Table S1: clinical characteristics of patients with colorectal tumours.
