# Supplementary material for: Overexpression of IRS-4 Correlates with Procaspase 3 Levels in Tumoural Tissue of Patients with Colorectal Cancer
Source: J Oncol. 2018 Oct 16;2018:3812581. doi: 10.1155/2018/3812581 (PMC6206579; doi:10.1155/2018/3812581)
Supplement: Supplementary Materials — Table S1: clinical characteristics of patients with colorectal tumours. [file 3812581.f1.pdf]

Supplementary Material

Table S1: Clinical characteristics of patients with colorectal tumours

Patient	Age	Gender	Diagnostic	Location	TNM	Tumor grade
1	49	M	Adenocarcinoma	Descending colon	T2N0M0	Moderately
2	67	F	Adenocarcinoma	Rectum	T1N0M0	Well
3	71	M	Adenocarcinoma	Ascending colon	T4bN1M0	Poorly
4	67	F	Adenocarcinoma	Sigmoid	T3aN1Mx	Poorly
5	60	M	Adenocarcinoma	Sigmoid	T3aN0Mx	Moderately
6	76	M	Adenocarcinoma	Ascending colon	T1N0Mx	Moderately
7	74	M	Adenocarcinoma	Rectum	T3cN1Mx	Poorly
8	58	M	Adenocarcinoma	Rectum	T1N0M0	Well
9	58	M	Tubulovillous adenomas	Rectum	TisN0Mx	Well
10	50	M	Tubulovillous adenomas	Transverse colon	TisN0M0	Well
11	48	F	Adenocarcinoma	Rectum	T3aN1Mx	Moderately
12	76	M	Adenocarcinoma	Sigmoid	T3dN2aM	Poorly
13	74	M	Adenocarcinoma	Transverse colon	T1N0Mx	Moderately
14	60	M	Adenocarcinoma	Rectum	T3aN1Mx	Moderately
15	77	M	Adenocarcinoma	Rectum	T3aN2Mx	Poorly
16	80	M	Adenocarcinoma	Ascending colon	T3aN0Mx	Moderately
17	66	M	Adenocarcinoma	Ascending colon	T3aN0Mx	Moderately
18	80	M	Adenocarcinoma	Sigmoid	T1N0Mx	Moderately
19	77	M	Adenocarcinoma	Sigmoid	T3aN1M1	Poorly
20	80	F	Adenocarcinoma	Ascending colon	T2N0M0	Moderately
